# Lipoprotein(a) As a Risk Factor in a Cohort of Hospitalised Cardiovascular Patients: A Retrospective Clinical Routine Data Analysis

**DOI:** 10.3390/jcm12093220

**Published:** 2023-04-29

**Authors:** David Šuran, Tadej Završnik, Peter Kokol, Marko Kokol, Andreja Sinkovič, Franjo Naji, Jernej Završnik, Helena Blažun Vošner, Vojko Kanič

**Affiliations:** 1 Department of Cardiology and Angiology, University Medical Centre Maribor, Ljubljanska ulica 5, 2000 Maribor, Slovenia; 2 Faculty of Medicine, University of Maribor, Taborska ulica 8, 2000 Maribor, Slovenia; 3 Faculty of Electrical Engineering and Computer Science, University of Maribor Koroška cesta 46, 2000 Maribor, Slovenia; 4 Semantika Research, Semantika d.o.o., Zagrebška ulica 40a, 2000 Maribor, Slovenia; 5 Department of Medical Intensive Care, University Medical Centre Maribor, Ljubljanska ulica 5, 2000 Maribor, Slovenia; 6 Community Healthcare Center Dr. Adolf Drolc Maribor, Ulica talcev 9, 2000 Maribor, Slovenia; 7 Alma Mater Europaea, Slovenska ulica 17, 2000 Maribor, Slovenia; 8 Faculty of Health and Social Sciences Slovenj Gradec, Glavni trg 1, 2380 Slovenj Gradec, Slovenia

**Keywords:** acute myocardial infarction, aortic valve stenosis, atherosclerosis, cardiovascular diseases, cardiovascular risk, ischemic heart disease, lipoprotein(a), postmenopausal women

## Abstract

Introduction: Lipoprotein(a) (Lp(a)) is a well-recognised risk factor for ischemic heart disease (IHD) and calcific aortic valve stenosis (AVS). Methods: A retrospective observational study of Lp(a) levels (mg/dL) in patients hospitalised for cardiovascular diseases (CVD) in our clinical routine was performed. The Lp(a)-associated risk of hospitalisation for IHD, AVS, and concomitant IHD/AVS versus other non-ischemic CVDs (oCVD group) was assessed by means of logistic regression. Results: In total of 11,767 adult patients, the association with Lp(a) was strongest in the IHD/AVS group (e^β^ = 1.010, *p* < 0.001), followed by the IHD (e^β^ = 1.008, *p* < 0.001) and AVS group (e^β^ = 1.004, *p* < 0.001). With increasing Lp(a) levels, the risk of IHD hospitalisation was higher compared with oCVD in women across all ages and in men aged ≤75 years. The risk of AVS hospitalisation was higher only in women aged ≤75 years (e^β^ = 1.010 in age < 60 years, e^β^ = 1.005 in age 60–75 years, *p* < 0.05). Conclusions: The Lp(a)-associated risk was highest for concomitant IHD/AVS hospitalisations. The differential impact of sex and age was most pronounced in the AVS group with an increased risk only in women aged ≤75 years.

## 1. Introduction

Lp(a) is a low-density lipoprotein (LDL)-like particle with an additional apolipoprotein(a) [apo(a)] covalently bound to the apolipoprotein B [1]. Its recognition goes back to 1963 with Berg, et al., while its proatherogenic and prothrombotic properties have been extensively studied afterwards [2,3].

In 2008, the Danish general population observational study demonstrated a log-linear relationship between Lp(a) level and the risk of ischemic heart disease (IHD) and acute myocardial infarction (AMI) [4]. The causal link was further confirmed by Mendelian association studies and metanalyses of observational studies [5,6,7,8]. Most recently, the analysis of the largest Lp(a) database to date (the national United Kingdom Biobank) including epidemiologic and Lp(a) genetic score results confirmed a linear increase in the risk of IHD across the Lp(a) distribution [9]. The EPIC-Norfolk observational trial further demonstrated an association of Lp(a) with peripheral arterial disease, while the link with stroke remains more controversial [10,11].

In 2013, a genome-wide association study implicated an association of a genetic variant in the apo(a) gene (*LPA*) and increased Lp(a) with aortic valve calcification and calcific aortic valve stenosis (AVS) [12]. The link was further confirmed by the large Danish observational and Mendelian randomisation study, demonstrating a stepwise increase in the risk of AVS with increasing Lp(a) and corresponding *LPA* genotypes [13]. Similarly, in the ASTRONOMER trial, patients with the highest Lp(a) presented with an accelerated AVS progression rate and the need for earlier surgical interventions [14,15].

In general, women have 5–10% higher Lp(a) levels than men [16,17,18,19]. In men, Lp(a) level remains relatively constant across the lifespan, whereas in women, a modest increase at menopause was found [20,21,22,23]. Few studies suggested also a lower risk of cardiovascular (CV) events associated with elevated Lp(a) in the elderly, which, however, remains controversial [21,24,25,26].

Most current Lp(a) knowledge relies on prospective observational and genetic trials, while little real-world data from clinical routine is available [27,28,29,30]. To acquire new knowledge from our everyday clinical practice, we performed an observational retrospective study of Lp(a) distribution in a cohort of hospitalised patients at our department. Since the data about the impact of sex and age are less consistent, we focused on the Lp(a)-associated risk of hospitalisation for IHD, AVS, and concomitant IHD/AVS in different sex and age groups.

## 2. Materials and Methods

### 2.1. Participants

In our hospital database, we retrospectively identified a cohort of patients aged ≥ 18 years who were hospitalised at the Department of Cardiology and Angiology, University Medical Centre Maribor, Slovenia for cardiovascular diseases (CVDs) with at least one available Lp(a) result obtained in the period 2000–2020 (inclusion criterion). Most Lp(a) values were collected in the years 2005–2008, where Lp(a) was part of the routine laboratory protocol on admission. Outside this time period, Lp(a) was measured occasionally if requested by the attending physician. Since the association of Lp(a) levels with IHD and AVS versus non-ischemic CVDs were studied, an exclusion criterion were hospitalisations for ischemic CVDs other than IHD (peripheral arterial disease and a history of ischemic stroke). Another exclusion criterion was treatment with inclisiran and monoclonal antibodies to proprotein convertase subtilisin/kexin type 9 (PCSK9) with a known significant Lp(a)-lowering effect [31,32,33]. In patients with more hospitalisations, only the data concerning initial hospitalisation were considered. In the case of more laboratory tests during the first visit, the median value was used for statistical analyses. The retrospective data analysis was in accordance with the Helsinki Declaration and was approved by the local Ethics Committee (UKC-MB-KME-8/23; 23 February 2023).

### 2.2. Definition of Studied Groups

The Tenth Revision of the International Classification of Diseases (ICD-10) diagnostic codes in the discharge report were used to stratify eligible patients into four diagnosis-based groups (ICD-10 codes in brackets): (i) IHD (I20–I25), (ii) AVS (I35.0, I35.2), (iii) concomitant AVS/IHD (I20–I25 and I35.0/I35.2), and (iv) the ‘other non-ischemic cardiovascular diseases’ (oCVD) group of patients admitted for diseases other than ischemic CVDs and AVS. The most frequent ICD-10 discharge diagnoses in the oCVD group were: I48 (atrial fibrillation and flutter), I10 (essential hypertension), I42.0 (dilated cardiomyopathy), I47.1 (supraventricular tachycardia), I34.0 (nonrheumatic mitral insufficiency), M54.6 (pain in thoracic spine).

To study the age- and sex-related impact of Lp(a) level on IHD, AVS and concomitant IHD/AVS hospitalisations, each diagnosis-based group (IHD, AVS, IHD/AVS, oCVD) was further divided into six age and sex subgroups (<60 years, 60–75 years, and >75 years). The age threshold of 60 years was chosen due to the known increase in Lp(a) levels in postmenopausal women, whereas another threshold of 75 years was used to study Lp(a) specifically in the elderly.

### 2.3. Statistical Analysis

A descriptive analysis of demographic and laboratory data on admission was performed. The normality of distribution was verified using the Kolmogorov-Smirnov test. Categorical and normally distributed laboratory variables of the IHD, AVS, and IHD/AVS groups were compared with the corresponding variables of the oCVD group using Fisher exact test and the independent samples *t*-test, as appropriate. Mann-Whitney test was used to compare non-normally distributed Lp(a) values and Lp(a) distribution between sexes in the matched age subgroups.

The risk of hospitalisation for IHD, AVS, and IHD/AVS versus oCVD, associated with increasing Lp(a) levels, was assessed by means of logistic regression. The exponential value of regression coefficient β (e^β^) represents an increase in the likelihood of hospitalisation for IHD, AVS, and IHD/AVS versus oCVD after an increase in Lp(a) level by 1.0 mg/dL. Based on the well-known association of Lp(a) specifically with ischemic CVDs and AVS, the oCVD group was used as a reference for comparisons, since healthy controls were not available [8,9,19,29]. The model was adjusted for age, sex, and other lipid parameters (LDL-cholesterol, HDL-cholesterol, triglycerides). In regression analysis, Lp(a) cholesterol (30% of Lp(a) mass) was subtracted from LDL-cholesterol to obtain corrected LDL values for an appropriate adjustment [34].

In each studied age and sex subgroup of the IHD, AVS, and IHD/AVS groups, the risk of hospitalisation associated with increasing Lp(a) levels was assessed by means of logistic regression, while sex- and age-matched oCVD subgroups were used as a reference for comparisons. The e^β^ represents an increase in the likelihood of hospitalisation in the studied subgroup versus matched oCVD subgroup after an increase in Lp(a) level by 1.0 mg/dL. The analysis was adjusted for LDL-cholesterol (corrected for Lp(a) cholesterol), HDL-cholesterol, and triglycerides.

All statistical analyses were performed by SPSS version 28 (IBM, Armonk, New York, NY, USA). A *p*-value < 0.05 was considered statistically significant.

### 2.4. Laboratory Data

Blood samples were obtained and analysed on the day of admission. Quantitative serum determination of Lp(a) was performed by a fully automated particle enhanced immunonephelometric Siemens N Latex assay (BN II System, Siemens Healthcare Diagnostics, Erlangen, Germany). The Lp(a) results were given in mass units (mg/L) [35]. Immunonephelometry detects aggregated polystyrene particles coated with specific antibodies to the analysed antigen using their ability to reflect light [36]. Since light scatter is measured, the light detector is oriented at an angle relative to the incident light source. The amount of light reaching the detector in immunonephelometry is directly proportional to the quantity of antigen analysed in the sample. The signal from samples is compared with a standardised calibrated concentration curve [37].

Triglycerides were measured the by the enzymatic method (Siemens Healthcare Diagnostics) and total serum cholesterol by the cholesterol esterase enzymatic assay (Siemens Healthcare Diagnostics). LDL- and HDL-cholesterol were measured by the homogenous direct method (Siemens Healthcare Diagnostics).

## 3. Results

A total of 11,767 patients were included into the study. Compared with the oCVD group, percentage of women was significantly lower in the IHD and IHD/AVS group (34.7% and 36.6% versus 47%, respectively, *p* < 0.001 for both comparisons), while sex distribution was similar in the AVS and oCVD groups. In the IHD, AVS, and IHD/AVS groups, patients were significantly older compared with the oCVD group (Table 1).

Laboratory parameters are presented in Table 1. Compared with the oCVD group, patients in the IHD and IHD/AVS groups presented with a significantly lower HDL-cholesterol (1.1 ± 0.3 and 1.1 ± 0.4 versus 1.2 ± 0.4 mmol/L, respectively), while LDL-cholesterol and triglycerides were higher in the IHD group (3.1 ± 1.1 versus 3.0 ± 1.1 mmol/L for LDL-cholesterol and 1.9 ± 1.5 versus 1.7 ± 1.3 mmol/L for triglycerides, *p* < 0.001 for both comparisons). The numbers of patients included into each of the four diagnosis-based groups (IHD, AVS, AVS/IHD, oCVD) are presented in the Supplementary Table S1.

In the IHD, AVS, and IHD/AVS groups, median Lp(a) levels were significantly higher compared with the oCVD group (12.0 mg/dL in the oCVD group, 15.0 mg/dL in the IHD and AVS groups, and 19.0 mg/dL in the IHD/AVS group, *p* < 0.001 for all comparisons) (Table 1). After stratification by sex and age, the lowest median level of Lp(a) was found in men aged < 60 years in the oCVD group (10.0 mg/dL) and the highest in women with concomitant IHD/AVS aged < 60 years (39.5 mg/L) (Table 2). Lp(a) levels were significantly higher in women compared with men in the IHD subgroup aged ≥ 60 years (*p* < 0.01, Z = 3.58) and in the oCVD subgroup aged ≤75 years (*p* = 0.01, Z = 2.54) (Supplementary Table S2).

To assess the Lp(a)-associated risk of hospitalisation for IHD, AVS, and IHD/AVS versus oCVD, an adjusted logistic regression analysis of Lp(a) as a continuous variable was performed. An adjusted regression analysis yielded the highest value of e^β^ for IHD/AVS (e^β^ = 1.010, *p* < 0.001) hospitalisation, followed by IHD (e^β^ = 1.008, *p* < 0.001) and AVS hospitalisation (e^β^ = 1.004, *p* < 0.001).

With increasing levels of Lp(a), the risk of IHD hospitalisation was higher compared with oCVD in men aged ≤75 years (e^β^ = 1.010 in age < 60 years; e^β^ = 1.008 in age 60–75 years, *p* < 0.001) and in women across all ages (e^β^ = 1.008, *p* < 0.001 in age ≤ 75 years; e^β^ = 1.006, *p* = 0.003 in age > 75 years) (Table 3).

With increasing levels of Lp(a), the risk of AVS hospitalisation was higher compared with oCVD in women aged ≤75 years (e^β^ = 1.010, *p* = 0.017 in age < 60 years; e^β^ = 1.005, *p* = 0.032 in age 60–75 years), while in men, the risk was similar to the oCVD group (Table 3).

With increasing levels of Lp(a), the risk of IHD/AVS hospitalisation was higher compared with oCVD in women across all ages (e^β^ = 1.023, *p* = 0.002 in age < 60 years, e^β^ = 1.011, *p* < 0.001 in age 60–75 years, and e^β^ = 1.010, *p* = 0.003 in age > 75 years), whereas in men, the risk was higher only in those aged 60–75 years (e^β^ = 1.011, *p* < 0.001) (Table 3).

## 4. Discussion

In our retrospective observational study of a cohort of hospitalised CVD patients, the association with increasing Lp(a) levels was strongest in the IHD/AVS group, followed by the IHD and AVS group, while sex- and age-related differences were highlighted. The Lp(a)-associated risk of IHD hospitalisation decreased in elderly men (>75 years), while it was less affected by age in women. The differential impact of sex and age was most pronounced in the AVS group with an increased Lp(a)-associated risk only in women aged ≤75 years.

A great amount of epidemiologic and genetic evidence confirmed Lp(a) as a causal risk factor for IHD and AVS [5,6,7,8,38,39,40,41]. However, the data comparing the strength of association of Lp(a) with the incidence of IHD and AVS are less clear. In the Danish general population studies, higher hazard ratios for AMI (3.6 and 3.7 for women and men, respectively) than for AVS (2.9 for both sexes together) were found in those with an Lp(a) > 95th percentile [4,13]. In contrast, a Mendelian randomization study based on the United Kingdom Biobank data reported a stronger association of *LPA* risk genotypes with AVS than IHD [42]. In our study, the association of increasing Lp(a) levels with AVS was weaker than with IHD hospitalisation. To our knowledge, our study is one of the first evaluating Lp(a) as a risk factor for concomitant IHD/AVS. The risk of concomitant IHD/AVS hospitalisation was higher than for IHD or AVS hospitalisation alone and was more consistent across age subgroups in women than in men. As the Lp(a) data in patients with concomitant AVS/IHD are lacking, further studies are needed to clarify the association better.

The impact of sex on Lp(a)-associated CV risk has already been demonstrated in some previous reports [20,24,43]. Lp(a) is on average higher in women than men, and a modest increase in Lp(a) has been demonstrated at menopause [18,20,21,22,23]. However, there are some conflicting data, whether the same Lp(a) level confers the same risk of CVD in both sexes [24,26,44,45]. In 1996, Sunayama S. et al. published one of the first reports suggesting sex differences in the Lp(a)-associated risk of coronary artery disease (CAD) [24]. In their study, the predictive value of Lp(a) for CAD began to decline 10 years earlier in women than in men [24]. In a study from the United States, among 3972 elderly adults (aged ≥ 65 years), an increased Lp(a) level was an independent predictor of stroke in men but not in women [26]. In the JUPITER trial cohort, a strong association between Lp(a) and CVD was demonstrated in men, while attenuated in women [43,46]. On the other hand, a recent large Danish general population study found that the same increase in Lp(a) level similarly affects CV risk in men and women aged >50 years [21]. In our study, IHD hospitalisation was associated with higher levels of Lp(a) in both sexes aged < 75 years, while no association was found in elderly men. The impact of sex was even more evident in the AVS group, where an increased Lp(a)-associated risk was demonstrated only in women.

Besides differential impact of sex, an impact of age on the CV risk associated with elevated Lp(a) is another controversial issue. In a study including Japanese-American men, the hazard ratios for an incident AMI in patients with an Lp(a) level > 20 mg/dL dropped form 2.5 in those aged < 60 years to only 1.2 in those aged >70 years [47]. In a population-based case-control study of women from the Stockholm area, the odds of CAD in the highest versus the lowest quartile of Lp(a) declined from 5.1 (95% CI, 1.4 to 18.4) in premenopausal to 2.4 (95% CI, 1.3 to 4.5) in postmenopausal women, respectively [25]. An attenuated impact of Lp(a) on CAD in the elderly was observed also in few other reports, however, it was not a universal finding [21,48,49]. In our cohort, the association of IHD, AVS, and IHD/AVS hospitalisations with higher levels of Lp(a) was weaker in advanced age (>75 years), while the impact of age was much more pronounced in men. In the IHD group, sex- and age-related differences might be explained by significantly higher Lp(a) levels in elderly women compared with elderly men, suggesting a higher Lp(a)-related IHD burden in women due to the postmenopausal increase in Lp(a). However, the reason for sex- and age-related differences in the AVS group with a similar Lp(a) distribution between sexes is less obvious. The Lp(a)-associated CV risk estimates seem to be masked by a more decisive risk factor like advanced age, which might be more apparent in men. In light of an inconsistency in presented data, sex- and age-related differences in the CV risk associated with elevated Lp(a) levels as well as the reason behind warrant further research.

Since new efficient Lp(a)-lowering drugs are being developed (antisense oligonucleotides, small interfering ribonucleic acid targeting apo(a) messenger RNA) and clinical outcome studies are awaited, the subgroups of patients with the largest expected treatment benefit need clarification in further research [31,32,33].

### Limitations

Our results should be interpreted in the context of certain limitations. The analysis was retrospective and relied on the reported ICD-10 diagnoses at hospital discharge given by the discharging physician. Due to the retrospective approach, a selection bias cannot be excluded. The number of women in the AVS/IHD group aged < 60 years was limited. The analysis included patients hospitalised for various CVDs, while no healthy controls were available.

In the regression analysis, results were adjusted for the impact of age, sex, and other lipids, but not for the potential influence of other CVD risk factors like smoking, arterial hypertension, diabetes, concomitant medications, and diet. Since more than 90% of Lp(a) variability was explained by genetics, diet and lifestyle interventions might not have significantly influenced our results [50].

## 5. Conclusions

In our retrospective observational study, Lp(a) was analysed as a risk factor in a cohort of patients hospitalised for IHD and AVS. An adjusted regression analysis yielded the strongest association of increasing Lp(a) levels with hospitalisations for concomitant IHD/AVS, followed by IHD and AVS. The Lp(a)-associated risk of IHD hospitalisation decreased in elderly men (>75 years), while it was less affected by age in women. In the AVS group, the differential impact of sex and age was even more pronounced with an increased Lp(a)-associated risk only in women aged ≤75 years. Further studies are needed to clarify the interaction of sex and age with the CV risk associated with elevated Lp(a) as well as the reasons behind.

## Figures and Tables

**Table 1:** Clinical and laboratory data of patients in the four diagnosis-based groups.

Parameters	Ischemic Heart Disease (*n* = 5554)	Aortic Valve Stenosis (*n* = 910)	Ischemic Heart Disease/Aortic Valve Stenosis (*n* = 486)	Other Non-Ischemic Cardiovascular Diseases (*n* = 4817)
Age (years), mean ± SD	64.1 ± 11.2 ***	68.6 ± 11.9 ***	71.8 ± 8.7 ***	61.1 ± 15.6
Women/Men (%)	34.7/65.3 ***	47.7/52.3	36.6/63.4 ***	47.0/53.0
Hemoglobin (g/L), mean ± SD	138.5 ± 16.5 *	135.0 ± 16.3 ***	133.5 ± 17.5 ***	138.0 ± 17.5
Thrombocytes (10^9^/L), mean ± SD	229.7 ± 72.3	218.7 ± 67.0 **	217.5 ± 70.2 **	229.2 ± 71
Urea (mmol/L), mean ± SD	7.5 ± 3.4 *	8.3 ± 3.8 ***	8.7 ± 4.1 ***	7.4 ± 4.3
Creatinine (mmol/L), mean ± SD	83.7 ± 65.8 ***	79.2 ± 65.8	86.4 ± 84.1 *	78.3 ± 73.1
Uric acid (μmol/L), mean ± SD	335.9 ± 104.8	350.3 ± 117.4	365.9 ± 117.3	335.8 ± 121.2
Total cholesterol (mmol/L), mean ± SD	5.0 ± 1.3	5.0 ± 1.3	4.7 ± 1.5 ***	4.9 ± 1.4
HDL-cholesterol (mmol/L), mean ± SD	1.1 ± 0.3 ***	1.2 ± 0.4	1.1 ± 0.4 ***	1.2 ± 0.4
LDL-cholesterol (mmol/L), mean ± SD	3.1 ± 1.1 ***	3.0 ± 1.2	2.9 ± 1.2	3.0 ± 1.1
Triglycerides (mmol/L), mean ± SD	1.9 ± 1.5 ***	1.5 ± 0.8 **	1.6 ± 1.1	1.7 ± 1.3
Lp(a) (mg/dL), median (5th–95th percentile)	15.0 (2.0–115.0) ***	15.0 (2.0–103.0) ***	19.0 (2.0–131.0) ***	12.0 (2.0–89.0)

Legend: All included patients were divided into four diagnosis-based groups according to the discharge diagnoses: aortic valve stenosis, ischemic heart disease, concomitant ischemic heart disease/aortic valve stenosis, and a group of patients hospitalised for other non-ischemic cardiovascular diseases (oCVD). HDL, high density lipoprotein; LDL, low-density lipoprotein; SD, standard deviation. Normally distributed laboratory parameters are presented as mean ± standard deviation (SD). Lp(a) values were not normally distributed and are presented as median values and 5th–95th percentiles. * *p* < 0,05; ** *p* < 0.01; *** *p* < 0.001 for comparison with the oCVD group.

**Table 2:** Lp(a) distribution (mg/dL, median and 5th–95th percentile) in the four diagnosis-based groups, stratified by age and sex.

	Median Lp(a) Level, mg/dL (5th–95th Percentile)		
	<60 Years	60–75 Years	>75 Years
**Ischemic heart disease**			
Men	15.0 (2.0–116.1)	14.5 (2.0–104.1)	14.0 (2.0–95.0)
Women, median value (5th–95th percentile)	17.0 (2.0–132.0)	16.0 (2.0–133.5)	17.5 (2.0–113.1)
**Aortic valve stenosis**			
Men, median value (5th–95th percentile)	13.0 (2.0–96.3)	14.0 (2.0–89.0)	14.0 (2.0–93.4)
Women, median value (5th–95th percentile)	18.0 (2.2–130.1)	18.0 (2.0–116.0)	13.0 (2.0–113.1)
**Ischemic heart disease/aortic valve stenosis**			
Men, median value (5th–95th percentile)	14.0 (2.0–156.5)	17.0 (2.0–125.0)	17.0 (2.0–149.7)
Women, median value (5th–95th percentile)	39.5 (1.0–135.0)	24.0 (2.1–149.2)	20.0 (2.0–121.5)
**Other non-ischemic cardiovascular diseases**			
Men	10.0 (2.0–84.4)	12.0 (2.0–83.2)	12.0 (2.0–96.0)
Women, median value (5th–95th percentile)	12.0 (2.0–92.0)	13.0 (2.0–92.0)	14.0 (2.0–95.0)

Legend: All included patients were divided into four diagnosis-based groups according to the discharge diagnosis: aortic valve stenosis, ischemic heart disease, concomitant aortic valve stenosis/ischemic heart disease, and a group of patients hospitalised for other non-ischemic cardiovascular diseases (oCVD). Patients in each of the four diagnosis-based groups were further divided into 3 age subgroups: < 60 years, 60–75 years, and >75 years. For each subgroup, median Lp(a) values and 5th–95th percentile (in brackets) were calculated. All Lp(a) values are expressed in mass units (mg/dL).

**Table 3:** Logistic regression analysis representing the strength of association of increasing Lp(a) levels (e^β^) with hospitalisations for IHD, AVS, and concomitant IHD/AVS, adjusted for LDL-cholesterol, HDL-cholesterol, and triglycerides.

Diagnosis	Age Group, Years	Exponential Value of β (e^β^) Men	Exponential Value of β (e^β^) Women
Ischemic heart disease	<60	1.010 ***	1.008 ***
	60–75	1.008 ***	1.008 ***
	>75	1.001	1.006 **
Aortic valve stenosis	<60	1.005	1.010 *
	60–75	1.005	1.005 *
	>75	1.002	1.002
Ischemic heart disease/ aortic valve stenosis	<60	1.008	1.023 **
	60–75	1.011 ***	1.011 ***
	>75	1.006	1.010 **

Legend: e^β^, exponential value of logistic regression coefficient; e^β^ represents an increase in the likelihood of hospitalisation for an observed diagnosis compared with ‘other non-ischemic cardiovascular diseases’ (oCVD) following an increase in the Lp(a) level by 1.0 mg/dL. * *p* < 0.05; ** *p* < 0.01; *** *p* < 0.001 for comparisons with the oCVD group.

## Data Availability

The data that support the findings of this study are available from the corresponding author upon a reasonable request. The data are not publicly available due to ethical concerns.

## Supplementary Materials

The following supporting information can be downloaded at: https://www.mdpi.com/article/10.3390/jcm12093220/s1, Table S1: Number of patients included into each of the four diagnosis-based groups, statified by age and gender, Table S2: Mann-Whitney test for gender differences in Lp(a) distribution in the four diagnosis-based groups, stratified by age.

## Abbreviations

AMI	Acute myocardial infarction
Apo(a)	Apolipoprotein(a)
AVS	Aortic valve stenosis
CVD	Cardiovascular disease(s)
EAS	European Atherosclerosis Society
ESC	European Society of Cardiology
ICD-10	International Classifications of Diseases, Tenth edition
IHD	Ischemic heart disease
Lp(a)	Lipoprotein(a)
*LPA*	A gene locus encoding apolipoprotein(a)
oCVD	Other cardiovascular diseases
OR(s)	Odds radio(s)
PCSK9	proprotein convertase subtilisin/kexin type 9
siRNA	Small interfering ribonucleic acid
