# Supplementary material for: Lipoprotein(a) As a Risk Factor in a Cohort of Hospitalised Cardiovascular Patients: A Retrospective Clinical Routine Data Analysis
Source: J Clin Med. 2023 Apr 29;12(9):3220. doi: 10.3390/jcm12093220 (PMC10178911; doi:10.3390/jcm12093220)
Supplement: Supplementary file 1 [file jcm-12-03220-s001.zip › jcm-2289425-supplementary.pdf]

Journal of
Clinical Medicine

MDPI

Supplementary Material

Table S1. Number of patients included into each of the four diagnosis-based groups, statified by age and gender.

**Aortic valve stenosis**	**<60 years**	**60–75 years**	**>75 years**
Men (number of patients)	154	217	105
Women, (number of patients)	42	211	181
**Aortic valve stenosis/ischemic heart disease**	**<60 years**	**60–75 years**	**>75 years**
Men (number of patients)	34	194	80
Women (number of patients)	8	81	89
**Ischemic heart disease**	**<60 years**	**60–75 years**	**>75 years**
Men (number of patients)	1177	1957	495
Women (number of patients)	499	930	496
**Other non-ischemic cardiovascular diseases**	**<60 years**	**60–75 years**	**>75 years**
Men (number of patients)	1275	936	340
Women (number of patients)	868	861	537

Legend: All included patients were divided into four diagnosis-based groups according to the discharge diagnoses: aortic valve stenosis, ischemic heart disease, concomitant aortic valve stenosis/ischemic heart disease, and a group of patients hospitalised for other non-ischemic cardiovascular diseases. Patients in each of the diagnosis-based groups were further divided into 3 age subgroups.

Table S2. Mann-Whitney test for gender differences in Lp(a) distribution in the four diagnosis-based groups, stratified by age.

**Diagnoses group /Age**	**<60 years**	**60–75 years**	**>75 years**
Ischemic heart disease	p=0.33<br>Z=– 0.97	p=0.00<br>Z= –3.58	p=0.00<br>Z= –3.19
Aortic valve stenosis	p=0.19<br>Z= –1.30	p=0.06<br>Z= –1.92	p=0.87<br>Z= –0.16
Ischemic heart disease/ aortic valve stenosis	p=0.04<br>Z= –2.02	p=0.08<br>Z= –1.76	p=0.35<br>Z= –0.93
Other non-ischemic cardiovascular diseases	p=0.02<br>Z= –2.36	p=0.01<br>Z= –2.54	p=0.15<br>Z= –1.44

Legend: All included patients were divided into four diagnosis-based groups according to the discharge diagnoses: aortic valve stenosis, ischemic heart disease, concomitant aortic valve stenosis/ischemic heart disease, and the group of patients hospitalised for other non-ischemic cardiovascular diseases (oCVD). Mann-Whitney test was used to compare Lp(a) levels between men and women in the four diagnosis-based groups stratified by age. A p-value <0.05 was considered statistically significant; Z, Z-score. A negative Z score and a p-value <0.05 defined higher Lp(a) values in women compared with men in the observed age subgroup.
